# Risk of Infection with Methotrexate Therapy in Inflammatory Diseases: A Systematic Review and Meta-Analysis

**DOI:** 10.3390/jcm8010015

**Published:** 2018-12-21

**Authors:** Ammar Ibrahim, Mohammed Ahmed, Richard Conway, John J. Carey

**Affiliations:** 1 Department of Medicine, National University of Ireland Galway, Galway, Ireland; jamaleldeen@gmail.com; 2 Department of Rheumatic Diseases, St. James’s University Hospital, Dublin, Ireland; drrichardconway@gmail.com; 3 Department of Rheumatic Diseases, Galway University Hospitals, Galway, Ireland; john.j.carey@nuigalway.ie

**Keywords:** methotrexate, infections and arthritis, inflammation, DMARDs

## Abstract

The aim of this study was to determine the risk of infection in adults with inflammatory rheumatic diseases (IRDs) treated with methotrexate. We performed a systematic review and meta-analysis of randomized controlled trials (RCTs) assessing methotrexate versus placebo in adults using MEDLINE, EMBASE, and CENTRAL databases from 1980 to August 2017. The primary outcome was the risk of infection associated with methotrexate therapy. We chose a random effect model to summarize adverse event outcomes as risk ratios (RRs) and related 95% confidence intervals (95% CI). Twelve RCTs (total patients 1146) met the inclusion criteria for our main analysis, and ten for risk of serious infection (total patients 906). Overall, methotrexate was associated with increased risk of infection in rheumatoid arthritis (RA) (RR: 1.25; 95% CI, 1.01–1.56; *p* = 0.04; *I*^2^ = 0%), but not in other non-RA IRD populations. There was no increased risk of total infections (RR: 1.14; 95% CI, 0.98–1.34; *p* = 0.10; *I*^2^ = 0%) or serious infections (RR: 0.76; 95% CI, 0.11–5.15; *p* = 0.78; *I*^2^ = 0%) in all included IRDs. Conclusively, methotrexate use in IRDs is associated with a higher risk of all infections in RA, but not in other non-RA (IRD) populations. There is no increased risk of serious infections.

## 1. Introduction

Methotrexate is a commonly prescribed medication which primarily inhibits DNA synthesis [1,2]. It is highly efficacious and is thus the anchor therapy in the management of rheumatoid arthritis (RA) and a primary therapeutic choice in many other inflammatory and rheumatic conditions [3,4,5,6,7]. Serious side effects have been ascribed to methotrexate therapy including bone marrow suppression, pulmonary disease, liver fibrosis, and infection [8,9,10,11]. Recent studies determined that the risk of pulmonary disease and liver fibrosis is lower than previously believed [8,9,10,11].

Studies report an association of infections with specific rheumatic conditions such as RA, or with immune-suppressant medications used to treat these conditions, which may lead to poorer outcomes or death [12,13,14,15]. This may be true for some therapies, particularly biologic medications [16]. A recent review of multiple RA cohorts showed rates of hospitalized infection between 1.14 and 1.62 per 100 patients per annum [17]. Infection is the third leading cause of death in RA populations [18]. The risk of infectious adverse events with methotrexate therapy in inflammatory rheumatic diseases (IRDs) is unknown, as published studies report inconsistent results [12,19,20,21,22,23]. Some observational studies show that methotrexate increases the risk [19,20,21], while others do not [12,22,23]. Recent meta-analyses show an increased risk of infectious lung disease in RA [8], but not in non-RA IRD populations [24]. Validating and quantifying the risk is critical to support appropriate decision-making in clinical practice. Therefore, we undertook a systematic review and meta-analysis to clarify the risk of infection, serious infection, and death from infection in patients treated with methotrexate for inflammatory rheumatic diseases (IRDs).

## 2. Materials and Methods

### 2.1. Data Sources and Searches

A systematic search of the English language literature from 1980 to August 2017 was performed using the following three major databases: MEDLINE, EMBASE, and CENTRAL. A search was also performed for previous reviews and meta-analyses, and the bibliographies of all included studies. Multiple search terms related to the population of relevant inflammatory rheumatic diseases (IRDs) and the intervention “methotrexate” were used and were linked with appropriate Boolean operators such as “OR” and “AND”. The detailed search strategies for the databases are provided in the supplement Table S1.

### 2.2. Study Selection

An initial screen for the eligibility of retrieved articles was performed based on the title and abstract in duplicate. Subsequently, full text articles considered for inclusion were retrieved and examined by two separate authors (A.I. and M.A.) independently and in duplicate, using pre-specified inclusion and exclusion criteria.

The inclusion criteria were (1) randomized controlled trials of adult human subjects (≥18 years of age) with rheumatic arthritic conditions including osteoarthritis, inflammatory connective tissue diseases, and inflammatory diseases that can overlap with rheumatic conditions, specifically inflammatory bowel disease and psoriasis; (2) published in the English language; (3) studies that had at least two arms, with one arm receiving methotrexate alone, and another arm receiving placebo alone or some other intervention not known to increase infection risk; (4) studies of at least 12 weeks duration but without a sample size restriction; and (5) studies that assessed modern conventional doses of methotrexate ranging between 2.5 and 25 mg per week administered weekly by oral, subcutaneous, or intramuscular routes.

The exclusion criteria were (1) nonrandomized or observational studies; (2) studies reported in a non-English language; (3) trials that did not clearly report infectious adverse events; (4) studies that assessed high-dose methotrexate (>25 mg per week); (5) studies using methotrexate in combination with other immunosuppressive medication such as long-term corticosteroids, biologic or non-biologic disease-modifying anti-rheumatic drugs (DMARDs); and (6) studies comparing methotrexate to other immunosuppressive medications. We did not exclude trials allowing non-immunosuppressive stable concomitant drug therapy, such as paracetamol or non-steroidal anti-inflammatory drugs (NSAIDs), or studies including short-term rescue corticosteroid therapy (in a dose of less than 15 mg per day) for treating disease flares.

### 2.3. Data Extraction and Quality Assessment

Data were extracted independently and in duplicate by two authors (A.I. and M.A.), utilizing a form developed by The Cochrane Consumers and Communication Group (2016). Both authors (A.I. and M.A.) independently assessed the methodological quality of randomized controlled trials (RCTs) within and across studies using The Cochrane Collaboration’s risk of bias assessment tool as recommended by The Cochrane Handbook of Systematic Reviews of Interventions [25]. Disagreement in data extraction and risk of bias assessment between the two authors was resolved by discussion. Agreement between the two reviewers (A.I., M.A.) on study inclusion and quality assessment was assessed by means of the kappa statistic.

### 2.4. Data Synthesis and Analysis

We assessed the risk of infectious events utilizing extracted dichotomous data of either infection or none. A serious infection was predefined as one requiring hospital admission or intravenous antibiotic administration. If trials did not report a definition of serious infection, we extracted adverse events reported by authors as “serious infection” or “severe infection” and analyzed both as serious infectious adverse events. We calculated the risk ratio (RR) of infection and presented results with their related 95% confidence intervals (CI) as forest plots using a random effect model that utilized the Mantle–Haenzsel statistical method. The level of significance set in our analysis was *p* = 0.05. Statistical heterogeneity among included studies was assessed by means of the *I*^2^ statistic. We classified heterogeneity into three main groups (considerable heterogeneity if *I*^2^ > 75%, significant if >50%, or low if <50%) in accordance with the general guidance provided in The Cochrane Handbook of Systematic Reviews of Interventions [25]. Publication bias was assessed using funnel plots. All analyses were performed using Revman (Version 5.3, The Nordic Cochrane Centre, The Cochrane Collaboration, Copenhagen, Denmark) [26]. Pre-specified subgroup analyses were performed to explore differences in infectious adverse events among various conditions (IRDs), methotrexate doses (< or >15 mg weekly), and study sizes (< or >100 participants). The attributable risk of infection and quality of evidence were assessed in accordance with the GRADE quality of evidence assessment process utilizing GRADE Pro GDT software.

### 2.5. Management of Missing Data

We chose to review and analyze the published data of relevant studies. We did not attempt to contact study authors where data were missing or not sufficiently reported.

## 3. Results

### 3.1. Literature Search

**Figure 1:**
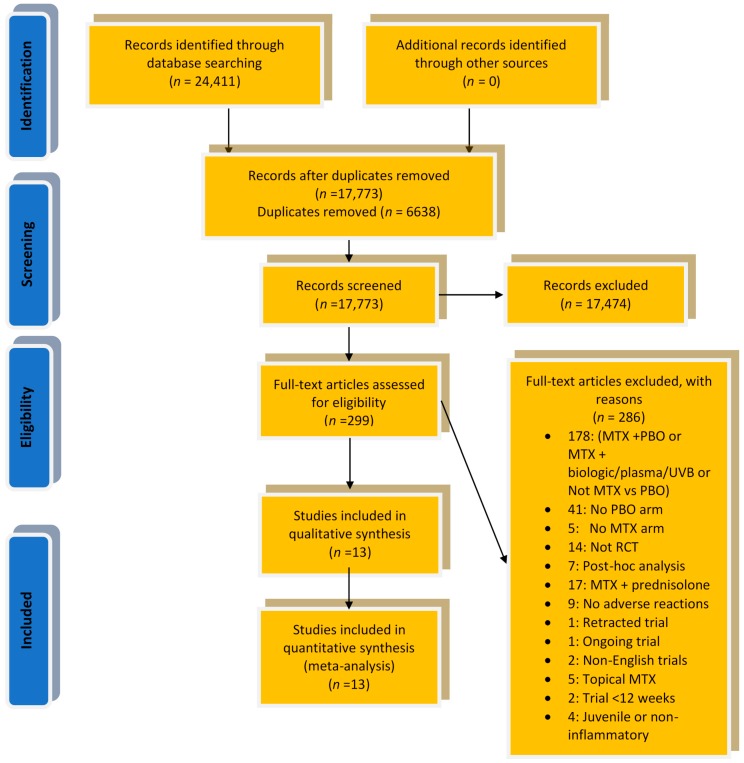
PRISMA flow diagram: Risk of infection of methotrexate therapy in inflammatory diseases (abbreviations: MTX—Methotrexate, PBO—Placebo, RCT—Randomized Controlled trial, UVB: Ultra violet B).

Our initial literature search retrieved 24,411 records—17,773 after removal of duplicates. Screening of the titles and abstracts resulted in the further exclusion of 17,473 articles, leaving 299 articles deemed suitable for a secondary eligibility assessment through a secondary detailed review of the full-text articles. This examination led to the exclusion of a further 286 records, leaving 13 for inclusion in our final review and analysis. Based on our eligibility criteria, the inflammatory rheumatic conditions (IRDs) that were included in this review were rheumatoid arthritis (RA), psoriasis, psoriatic arthritis (PsA), ankylosing spondylitis (AS), systemic sclerosis, and Crohn’s disease. All other retrieved IRD trials did not meet our pre-specified inclusion and exclusion criteria. The agreement between reviewers for study inclusion was excellent (kappa statistic 0.958). Details of the search results, study selection and review reporting are shown below in the PRISMA flow diagram (Figure 1) and PRISMA checklist (Table S2) in the supplement.

### 3.2. Study Characteristics

Of the trials included in this review, 12 assessed the risk of infection of methotrexate versus placebo, enrolling 1146 participants, and 10 assessed the risk of serious infection, enrolling 906 participants. Serious infectious adverse events were extracted based on trial reported terms “serious infection” or “severe infection”, but in most of these reports a definition of “serious” or “severe” infection was not stated. Trial durations ranged between 3 and 24 months with an enrolled number of participants of 18 to 482. The included studies comprised five different diseases (IRDs): five trials assessed participants with psoriasis or psoriatic arthritis (PsA), three assessed RA, two assessed systemic sclerosis, two assessed ankylosing spondylitis (AS), and one assessed Crohn’s disease. The range of mean ages of participants across the included trials was 33 to 59 years. All trials assessed a weekly methotrexate dose that ranged between 5 mg and 25 mg, with three trials assessing intra-muscular therapy and the remaining ten trials assessing oral therapy. The overall risk of bias across studies was deemed low. Only three trials were considered at risk of bias, with a “high risk” decision made across more than one domain (Figures S1 and S2 in Supplement). The agreement between reviewers for quality assessment was very good (kappa statistic 0.826). Overall, while there was heterogeneity between studies as noted above with different diseases, agents, and dosing regimens, the *I*^2^ value was <10% for all subsequent analyses. Details of the study characteristics are shown below in Table 1.

### 3.3. Risk of Infectious Adverse Events and Related Subgroup Analyses

**Figure 2:**
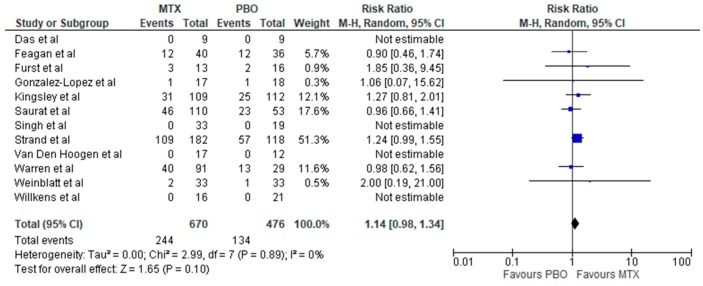
Forest plot: Risk of infection of methotrexate therapy in inflammatory diseases (abbreviations: MTX—methotrexate, PBO—placebo) (Studies included: [27,28,29,30,31,33,34,35,36,37,38,39]).

**Figure 3:**
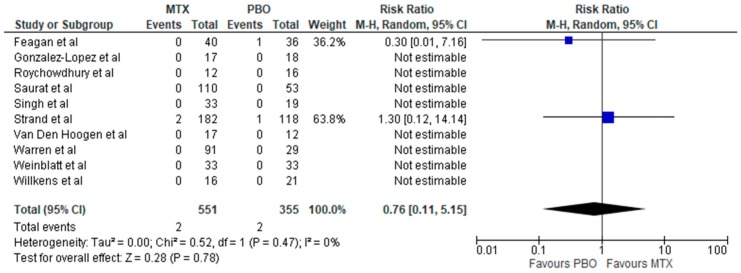
Forest plot: risk of serious infection of MTX vs PBO in inflammatory diseases (Studies included: [28,30,32,33,34,35,36,37,38,39]).

**Figure 4:**
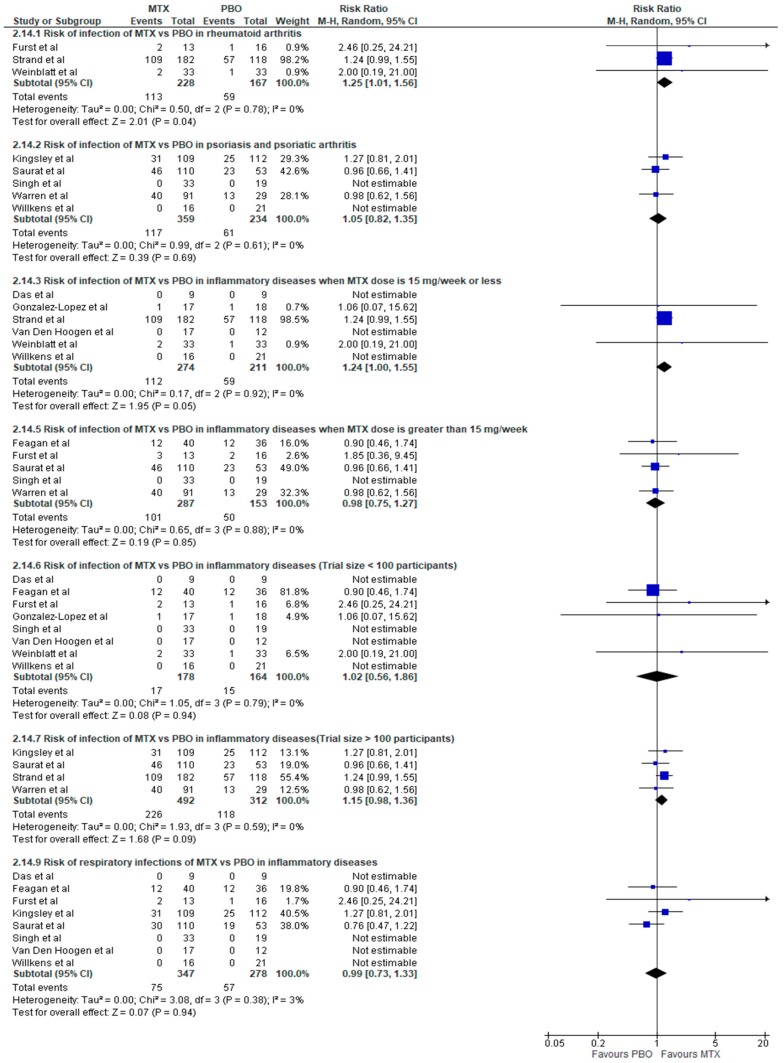
Forest plot: Subgroup analyses in risk of infection with methotrexate therapy in inflammatory diseases (Studies included: [27,28,29,30,31,33,34,35,36,37,38,39]).

**Figure 5:**
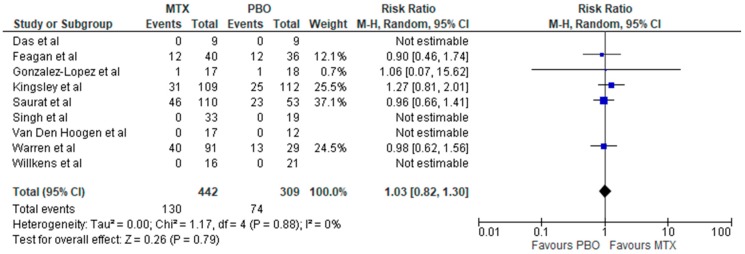
Forest plot: Risk of infection of methotrexate therapy in non-RA inflammatory diseases. (Studies included: [27,28,30,31,33,34,36,37,39]).

Overall, there was no increased risk of total and serious infection associated with methotrexate therapy in inflammatory rheumatic diseases (IRDs) with relative risks of RR: 1.14 (95% CI, 0.98–1.34; *p* = 0.10; *I*^2^ = 0%) and RR: 0.76 (95% CI, 0.11–5.15; *p* = 0.78; *I*^2^ = 0%), respectively (Figure 2 and Figure 3)**.** Methotrexate use compared to placebo was associated with increased risk of infection in RA (RR: 1.25; 95% CI, 1.01–1.56; *p* = 0.04; *I*^2^ = 0%) (Figure 4), but not in other non-RA IRD populations (RR: 1.03; 95% CI, 0.82–1.30; *p* = 0.79; *I*^2^ = 0%) (Figure 5). It was not possible to calculate the risk of death secondary to infection or provide a breakdown of infectious events by disease severity, and the only subtype of infection assessed was respiratory infections, due to a lack of sufficient published data in trial reports. There was no increased risk of respiratory infections (RR: 0.99; 95% CI, 0.73–1.33; *p* = 0.94; *I*^2^ = 3%) (Figure 4).

### 3.4. Further Subgroup Analyses

Further analysis of the risk of infection in inflammatory rheumatic diseases (IRDs) was performed on subgroups based on individual diseases, dose of methotrexate (< or >15 mg weekly), and size of trials (Figure 4). The analysis evaluating the dose showed an increased risk of infection with participants taking doses of 15 mg per week (RR: 1.24; 95% CI, 1.00–1.55; *p* = 0.05; *I*^2^ = 0%), but not with those on higher doses (RR: 0.98; 95% CI, 0.75–1.27; *p* = 0.85; *I*^2^ = 0%) (Figure 4). Methotrexate use was not associated with increased risk of infection in the subgroup of trials that had more than 100 participants (RR: 1.15; 95% CI, 0.98–1.36; *p* = 0.09; *I*^2^ = 0%) or in those with less than 100 participants (RR: 1.02; 95% CI, 0.56–1.86; *p* = 0.94; *I*^2^ = 0%) (Figure 4).

### 3.5. Additional Analyses

The absolute (excess) risk analysis of infection attributable to methotrexate therapy versus placebo was generated using Grade Pro GDT software. This showed that 88 more patients with RA treated with methotrexate per 1000 will develop an infection compared to placebo (95% CI, from 4 more to 198 more), while only 7 additional infectious adverse events per 1000 are attributed to methotrexate therapy in non-RA IRD patients (95% CI, from 43 fewer to 72 more). Funnel plot analysis of the risk of infection with methotrexate in inflammatory rheumatic diseases (IRDs) showed no evidence of a publication bias (Figure S3 in supplement). The overall quality of evidence synthesized from this review for evaluating the primary outcome as generated by the GRADE Pro GDT online software was moderate (Table S3 in the supplement).

## 4. Discussion

We report here the infectious risk associated with methotrexate compared to placebo in inflammatory rheumatic diseases (IRDs) including rheumatoid arthritis (RA), psoriasis and psoriatic arthritis (PsA), ankylosing spondylitis (AS), systemic sclerosis (SSc), and Crohn’s disease. In this study of 13 clinical trials, we found a small but significant increased risk of infection in RA, but not in other non-RA populations. There was no increased risk of total or serious infections in all inflammatory rheumatic diseases (IRDs) included in this review. In predefined analyses, we found an increased risk of infection in lower-dose methotrexate; further analyses showed that the result is due to the increased risk in RA populations rather than a dosing effect of methotrexate.

Infections are problematic for patients with inflammatory arthritis and related diseases [40], in part due to the resulting increase in morbidity and mortality [41,42], and also in trying to decide whether these represent a complication of their underlying illness, a complication of their immunosuppressive therapy, or an unrelated event. In clinical practice, it can be difficult to distinguish which of these processes is at play. Relationships can be complex, and even paradoxical, as seen with biologic drugs whereby there is an overall increased risk of infection [16,43] but perhaps a lower mortality from infection [44]. Patients with rheumatic disease experiencing an acute infection are often encountered in primary care, or general hospital settings, and are hence managed by non-rheumatologists [45,46]. Knowledge of infection risk with methotrexate therapy is essential for these practitioners to aid in the decision to withhold, continue, or restart effective treatment, such as methotrexate.

Observational studies of methotrexate use and infection risk report inconsistent results [12,19,20,21,23,47,48,49,50], with several studies showing no association [12,23,47,50,51], while others report an increased risk [19,20,21,48,49,52,53,54,55]. Recent large meta-analyses show no major difference in the risk of serious infections between biologics and traditional DMARDs including methotrexate therapy, and no impact from the concomitant use of methotrexate in rheumatic diseases [16,56]. These examined trials comparing methotrexate to other DMARDs, including biologics, making a true assessment of the risk difficult. Our study findings excluded trials with other medications known to increase the risk of infection, and are in agreement with prior publications [8,24]. These results should provide some reassurance to patients and practitioners.

Previous publications suggest that patients with RA who are prescribed methotrexate may be at higher risk of respiratory infection [8], but not patients with other inflammatory diseases [24]. In this study we found that the risk of all infections was significant in RA and low-dose methotrexate. The paradoxical effect seen with low-dose methotrexate was a surprise. However, further analyses showed that this effect was due to the confounding of the presence of RA. No increased risk was seen after adjustment for the presence of RA. Studies in RA are perhaps more complex than other diseases as RA patients are at increased risk of infection [48,49,57], which may lead to an overestimation of the risk of infectious outcomes related to treatment. Additionally, differentiating rheumatoid-related pneumonitis and pulmonary disease from an infection can be difficult in practice, unlike for other inflammatory disorders such as psoriatic arthritis (PsA), and ankylosing spondylitis (AS) [8,24,57]. The greater susceptibility to infection in RA can be explained by multiple factors including innate and adaptive immunological dysfunctions, chronic immunocompromising co-morbidities, older age population in RA, use of immune-suppressive drugs, interestingly, and bronchiectasis related to pulmonary involvement [8,57]. In our study, RA trials had a significant weight on the meta-analysis of studies, particularly among those with methotrexate doses of <15 mg per week. It appears that methotrexate use in RA is associated with a higher risk of infection.

A clear understanding of the benefits, risks, and cost of interventions is critical for medical practitioners and patients. Methotrexate is a drug of a relatively low cost with favorable clinical effectiveness in many inflammatory rheumatic diseases (IRDs) [58]. A recent network meta-analysis demonstrated the remarkable clinical efficacy of methotrexate therapy in rheumatoid arthritis following the examination of 158 clinical trials [3]. However, few specifically assessed the safety profile of methotrexate therapy [8,11,24,59] or expanded their population to multiple diseases [60,61,62]. A limited number evaluated the evidence pertaining to the risk of infection and serious infection [8,16,59,63]. Such studies are critically important since recent reviews have shown that the perceived risk of adverse events may be significantly lower than expected [8,11,24].

Our pre-specified analyses including the risk of total and respiratory infections are in agreement with other meta-analyses [8,24,59,63]. Recent evidence from large meta-analyses found no difference in the risk of serious infection between methotrexate naïve and experienced patients treated with biologic therapies [16,56]. Similarly, a recent review reported no significant increased risk of severe infections with methotrexate therapy [63]. We believe that the perceived risk of infectious adverse events related to methotrexate therapy in inflammatory diseases is probably lower than previously thought, supported by the evidence from this study. It may not always be appropriate, therefore, to withhold the drug, or switch to alternative DMARDs, where the actual culprit is the disease itself or an unrelated infection rather than its therapy.

Our study has several strengths. It resolves some of the inconsistency of results generated by multiple observational studies and clinical trials, and supports the evidence suggested by recent meta-analyses [8,16,24,56,59,63]. We captured evidence among a wider and more heterogeneous population than some prior studies limited to a single disease. The evidence is derived from multiple quality-controlled trials (RCTs) utilizing an appropriate methodology. The risk of publication bias was assessed and deemed to be low, while the overall quality of the review was moderate as based on a GRADES working group assessment. Our findings should provide some reassurance to clinicians and patients concerned about the risk of infection with this medication.

### Limitations

These findings should be interpreted judiciously for several reasons. We restricted our search to trials published in the English language only. Others have suggested that this may have minimal effects on the meta-analysis results [64,65], unless there are multiple large trials in another language with very different findings. We assumed in our study that outcomes assessed were similarly defined by all clinical trials (as in serious infections) because of the lack of detailed definitions in study reports. The effect of variability (if present) in outcome definitions on the quality of evidence generated is unknown. We only used published data, as we do not have access to patient-level data from these studies (some were quite old). This limited our analyses of some outcomes such as type of infection, breakdown of incident infection by disease severity, and risk of death due to infection. There was substantial attrition noted in some included clinical trials. We evaluated a subset of five diseases encountered in clinical practice, while remaining trials in IRDs were excluded based on our pre-specified eligibility criteria limiting generalizability of evidence. Exclusion of trials with insufficiently reported adverse events is another potential source of bias. The overall relatively small final sample size in this study in comparison to recently published meta-analyses is due to the restricted comparison of methotrexate monotherapy to placebo, an added factor that could affect the applicability of evidence generated in this review. However, it is these small multiple studies that meta-analysis may be most appropriate for, or where there is significant variation in conclusions in prior publications. Methodological limitations associated with meta-analyses of published RCTs are encountered such as the under-reporting of adverse events, incomplete and improperly reported rare events, and statistical approaches to dealing with zero events [25,66,67]. We have a small number of studies, some with little representation, and so factors such as population demographics and differences in drug metabolism between individuals and trials could alter medication response relationships and affect our results [68]. Finally, we did not include studies with other DMARDs, and selection bias is inherent in all clinical trials, perhaps limiting the external validity.

## 5. Conclusions

Methotrexate use in inflammatory rheumatic diseases (IRDs) included in this review is associated with a higher risk of all infections in RA, but not in other non-RA IRD populations. There is no increased risk of serious infections. A limited number of large high-quality studies are available to support these findings.

## Figures and Tables

**Table 1:** Characteristics of included studies on the risk of infection from methotrexate therapy in inflammatory diseases.

Study	Sample Size	Average Age (y)	Gender Female (%)	Trial Duration (Weeks)	Inflammatory Disease	Severity of Disease	Dose of MTX Assessed (mg/weeks)	Route of Administration	Risk of Bias ^1^
Das et al., 2005 [27]	18	33	16	72	SSc ^2^	Not specified	15	PO ^3^	Serious
Feagan et al., 2000 [28]	76	33	60	40	Crohn’s	Chronic active Crohn’s	15	IM ^4^	Low
Furst et al., 1989 [29]	46	55	63	18	RA ^5^	Not specified	7.5–15	PO	Low
Gonzalez-Lopez et al., 2004 [30]	35	35	31	24	AS ^6^	Active AS	7.5	PO	Low
Kingsley et al., 2012 [31]	221	48	44	24	PsA ^7^	Not specified	7.5	PO	Low
Roychowdhury et al., 2002 [32]	30	44	13	24	AS	Severe active AS	10	PO	Serious
Saurat et al., 2008 [33]	271	42	34	16	Psoriasis	Moderate to severe psoriasis	7.5	PO	Low
Singh et al., 2015 [34]	81	39	Not specified	12	Psoriasis	Severe psoriasis	>15	PO	Serious
Strand et al., 1999 [35]	482	54	73	52	RA	Active RA	7.5	PO	Low
Van Den Hoogen et al., 1996 [36]	29	54	71	24	SSc	Not specified (duration less than 3 years)	15	IM	Low
Warren et al., 2017 [37]	120	45	21	16	Psoriasis	Moderate to severe psoriasis	17.5	IM	Low
Weinblat et al., 1985 [38]	35	59	71	24	RA	Not specified	7.5–15	PO	Low
Willkens et al., 1984 [39]	37	45	59	12	PsA	Not specified	5	PO	Low

^1^: The Cochrane Risk of Bias Tool, ^2^: Systemic sclerosis, ^3^: Oral route, ^4^: Intramuscular route, ^5^: Rheumatoid arthritis, ^6^: Ankylosing spondylitis, ^7^: Psoriatic arthritis.

## Supplementary Materials

The following are available online: http://www.mdpi.com/2077-0383/8/1/15/s1, Table S1: Search strategy, Table S2: PRISMA checklist, Table S3: Summary of Findings and Quality of Evidence of the Primary Outcome, RA subgroup & Respiratory Infection According to GRADE Working Group Assessment Recommendations (GRADE pro GDT), Figure S1: Risk of bias graph, Figure S2: Risk of bias summary, Figure S3: Funnel plot to assess publication bias.
