# Supplementary material for: Risk of Infection with Methotrexate Therapy in Inflammatory Diseases: A Systematic Review and Meta-Analysis
Source: J Clin Med. 2018 Dec 21;8(1):15. doi: 10.3390/jcm8010015 (PMC6352130; doi:10.3390/jcm8010015)
Supplement: Supplementary file 1 [file jcm-08-00015-s001.pdf]

Table S1: Search strategy

Medline (Ovid) search

1. randomized controlled trial.pt.
2. controlled clinical trial.pt.
3. randomized.ab.
4. placebo.ab.
5. drug therapy.fs.
6. randomly.ab.
7. trial.ab.
8. groups.ab.
9. 1 or 2 or 3 or 4 or 5 or 6 or 7 or 8
10. (animals not (humans and animals)).sh.
11. 9 not 10
12. exp Methotrexate/
13. methotrexate.mp.
14. amethopterin.mp.
15. methotrexate hydrate.mp.
16. Methotrexate Sodium.mp.
17. Methotrexate, Dicesium Salt.mp.
18. Methotrexate, Disodium Salt.mp.
19. Methotrexate, Sodium Salt.mp.
20. Mexate.mp.
21. Anti-Rheumatic Agents.mp.
22. Anti-Rheumatic Drugs.mp.
23. exp Anti-Inflammatory Agents/ or exp Antirheumatic Agents/
24. Antirheumatic Disease-Modifying Second-Line Drugs.mp.
25. Antirheumatic Drugs.mp.
26. Antirheumatic Drugs, Disease-Modifying.mp.
27. DMARD.mp.
28. Disease-Modifying Antirheumatic Drugs.mp.
29. Disease-Modifying, Antirheumatic Second-Line Drugs.mp.
30. 12 or 13 or 14 or 15 or 16 or 17 or 18 or 19 or 20 or 21 or 22 or 23 or 24 or 25 or 26 or 27 or 28 or 29
31. exp Arthritis, Rheumatoid/
32. 11 and 30 and 31
33. exp Arthritis, Rheumatoid/
34. exp Arthritis, Psoriatic/
35. Arthritis, Psoriatic.mp.
36. Psoriasis, Arthritic.mp.
37. Psoriatic Arthropathy.mp.
38. palmoplantaris pustulosis.mp.
39. exp Psoriasis/
40. psoriasis.mp.
41. Pustulosis Palmaris et Plantaris.mp.
42. exp Spondylitis, Ankylosing/
43. ankylosing spondylarthritis.mp.
44. Ankylosing Spondylitis.mp.
45. ankylosing spondyloarthritis.mp.
46. Bechterew*.mp.
47. ankylosing spondyloarthritis$.mp.

48. psoriatic arthri*.mp.

49. psoriasis arthropath*.mp.

50. arthritis* psoriasis.mp.

51. Marie-Struempell Disease.mp.

52. rheumatoid spondylitis.mp.

53. Spondylitis Ankylopoietica.mp.

54. spondylarthritis ankylpoietica.mp.

55. Spondylitis, Ankylosing.mp.

56. Spondyloarthritis Ankylopoietica.mp.

57. exp Inflammatory Bowel Diseases/

58. Inflammatory Bowel Diseases.mp.

59. Bowel Diseases, Inflammatory.mp.

60. inflammatory bowel disease.mp.

61. exp Colitis, Ulcerative/

62. exp Crohn Disease/

63. Crohn Disease.mp.

64. exp Connective Tissue Diseases/

65. Connective Tissue Diseases.mp.

66. exp Collagen Diseases/

67. Collagen Diseases.mp.

68. exp Joint Diseases/

69. Joint Diseases.mp.

70. arthropathies.mp.

71. Arthrosis*.mp.

72. Arthropathy.mp.

73. exp Vasculitis/

74. Vasculitis.mp.

75. exp Lupus Vasculitis, Central Nervous System/

76. exp Anti-Neutrophil Cytoplasmic Antibody-Associated Vasculitis/

77. ANCA vasculitis.mp.

78. exp Systemic Vasculitis/

79. systemic vasculitis.mp.

80. exp Rheumatoid Vasculitis/

81. Rheumat* vasculitis.mp.

82. exp Retinal Vasculitis/

83. exp Vasculitis, Leukocytoclastic, Cutaneous/

84. exp Vasculitis, Central Nervous System/

85. Vasculitis.mp.

86. retinal vasculitis.mp. or Retinal Vasculitis/

87. leucocytoclastic vasculitis.mp.

88. Angiitis.mp.

89. exp Arteritis/

90. Arteritis.mp.

91. exp Polyarteritis Nodosa/

92. Periarteritis Nodosa.mp.

93. Polyarteritis Nodosa.mp.

94. exp Giant Cell Arteritis/

95. Aortic Arteritis, Giant Cell.mp.

96. Aortitis, Giant Cell.mp.

97. Arteritis, Giant Cell.mp.

98. Cranial Arteritis.mp.

99. Giant Cell Aortitis.mp.

100. Giant Cell Aortic Arteritis.mp.

101. Giant Cell Arteritis.mp.

102. Horton* Disease.mp.

103. Horton* arteritis.mp.

104. Horton* Giant Cell Arteritis.mp.

105. Temporal Arteritis.mp.

106. Cranial Arteritis.mp.

107. exp Autoimmune Diseases/

108. Autoimmune Disease*.mp.

109. Autoimmune Disease*.mp.

110. exp Polymyalgia Rheumatica/

111. Polymyalgia Rheumatica.mp.

112. Pseudopolyarthritis, Rhizomelic.mp.

113. Rheumatism, Extra-Articular.mp.

114. Rheumatism, Peri-Articular.mp.

115. exp Arthritis, Reactive/

116. Arthritis, Post-Infectious.mp.

117. Arthritis, Postinfectious.mp.

118. Arthritis, Reactive.mp.

119. Post-Infectious Arthritis.mp.

120. Postinfectious Arthritis.mp.

121. Reiter* Disease.mp.

122. Reiter* syndrome.mp.

123. exp Lupus Erythematosus, Systemic/

124. Lupus Erythematosus, Systemic.mp.

125. exp Lupus Erythematosus, Cutaneous/

126. exp Lupus Erythematosus, Discoid/

127. exp Lupus Nephritis/

128. Lupus.mp.

129. exp Lupus Vasculitis, Central Nervous System/

130. SLE.mp.

131. Libman-Sacks Disease.mp.

132. Lupus Erythematosus Disseminatus.mp.

133. Systemic Lupus Erythematosus.mp.

134. Lupus Erythematosus.mp.

135. exp Scleroderma, Systemic/

136. Scleroderma, Systemic.mp.

137. Systemic Scleroderma.mp.

138. Systemic Sclerosis.mp.

139. Aortitis Syndrome.mp.

140. exp Takayasu Arteritis/

141. Takayasu* Arteritis.mp.

142. Takayasu* Disease.mp.

143. Takayasu* syndrome.mp.

144. Pulseless Disease.mp.

145. exp Behcet Syndrome/

146. Behcet* Syndrome.mp.

147. Behcet* disease.mp.

148. Young Female Arteritis.mp.

149. Old Silk Route Disease.mp.

150. Triple Symptom Complex.mp.

151. Triple-Symptom Complex.mp.

152. exp Dermatomyositis/

153. Dermatomyositis.mp.

154. Polymyositis-Dermatomyositis.mp.

155. Dermatopolymyositis.mp.

156. exp Osteoarthritis/

157. Osteoarthritis.mp.

158. Osteoarthrosis.mp.

159. exp Chondrocalcinosis/

160. chondrocalcinosis.mp.

161. Calcium Pyrophosphate Deposition Disease.mp.

162. CPPD.mp.

163. Calcium Pyrophosphate Dihydrate Deposition.mp.

164. Pseudogout.mp.

165. exp Gout/

166. Gout.mp.

167. exp Crystal Arthropathies/

168. Crystal Arthritis.mp.

169. Crystalline Arthritis.mp.

170. Crystalline Arthropathies.mp.

171. exp Granulomatosis with Polyangiitis/

172. Granulomatosis with Polyangiitis.mp.

173. Wegener* Granulomatosis.mp.

174. Granulomatosis, Wegener*.mp.

175. Rheumatic disease*.mp.

176. exp Rheumatic Diseases/

177. 31 or 32 or 33 or 34 or 35 or 36 or 37 or 38 or 39 or 40 or 41 or 42 or 43 or 44 or 45 or 46 or 47 or 48 or 49 or 50 or 51 or 52 or 53 or 54 or 55 or 56 or 57 or 58 or 59 or 60 or 61 or 62 or 63 or 64 or 65 or 66 or 67 or 68 or 69 or 70 or 71 or 72 or 73 or 74 or 75 or 76 or 77 or 78 or 79 or 80 or 81 or 82 or 83 or 84 or 85 or 86 or 87 or 88 or 89 or 90 or 91 or 92 or 93 or 94 or 95 or 96 or 97 or 98 or 99 or 100 or 101 or 102 or 103 or 104 or 105 or 106 or 107 or 108 or 109 or 110 or 111 or 112 or 113 or 114 or 115 or 116 or 117 or 118 or 119 or 120 or 121 or 122 or 123 or 124 or 125 or 126 or 127 or 128 or 129 or 130 or 131 or 132 or 133 or 134 or 135 or 136 or 137 or 138 or 139 or 140 or 141 or 142 or 143 or 144 or 145 or 146 or 147 or 148 or 149 or 150 or 151 or 152 or 153 or 154 or 155 or 156 or 157 or 159 or 160 or 161 or 162 or 163 or 164 or 165 or 166 or 167 or 168 or 169 or 170 or 171 or 172 or 173 or 174 or 175 or 176

178. 11 and 30 and 177

Table S2: PRISMA CHECKLIST

Section/topic	#	Checklist item	Reported on page #
**TITLE**			
Title	1	Identify the report as a systematic review, meta-analysis, or both.	Page 1 (article)
**ABSTRACT**			
Structured summary	2	Provide a structured summary including, as applicable: background; objectives; data sources; study eligibility criteria, participants, and interventions; study appraisal and synthesis methods; results; limitations; conclusions and implications of key findings; systematic review registration number.	Page 2 (article)
**INTRODUCTION**			
Rationale	3	Describe the rationale for the review in the context of what is already known.	Page 3 (article)
Objectives	4	Provide an explicit statement of questions being addressed with reference to participants, interventions, comparisons, outcomes, and study design (PICOS).	Page 3(article)
**METHODS**			
Protocol and registration	5	Indicate if a review protocol exists, if and where it can be accessed (e.g., Web address), and, if available, provide registration information including registration number.	-Protocol (designed a priori) -Not published
Eligibility criteria	6	Specify study characteristics (e.g., PICOS, length of follow-up) and report characteristics (e.g., years considered, language, publication status) used as criteria for eligibility, giving rationale.	Pages 5 (article)
Information sources	7	Describe all information sources (e.g., databases with dates of coverage, contact with study authors to identify additional studies) in the search and date last searched.	Page 4 (article)
Search	8	Present full electronic search strategy for at least one database, including any limits used, such that it could be repeated.	Online only supplement (1)
Study selection	9	State the process for selecting studies (i.e., screening, eligibility, included in systematic review, and, if applicable, included in the meta-analysis).	Pages 4,5 & 8(article)
Data collection process	10	Describe method of data extraction from reports (e.g., piloted forms, independently, in duplicate) and any processes for obtaining and confirming data from investigators.	Pages 5&6 (article)
Data items	11	List and define all variables for which data were sought (e.g., PICOS, funding sources) and any assumptions and simplifications made.	Page 6 (article)
Risk of bias in individual studies	12	Describe methods used for assessing risk of bias of individual studies (including specification of whether this was done at the study or outcome level), and how this information is to be used in any data synthesis.	Page 5 (article)
Summary measures	13	State the principal summary measures (e.g., risk ratio, difference in means).	Page 6 (article)
Synthesis of results	14	Describe the methods of handling data and combining results of studies, if done, including measures of consistency (e.g., I^2) for each meta-analysis.	Page 6 (article)

Section/topic	#	Checklist item	Reported on page #
Risk of bias across studies	15	Specify any assessment of risk of bias that may affect the cumulative evidence (e.g., publication bias, selective reporting within studies).	Page 5 (article)
Additional analyses	16	Describe methods of additional analyses (e.g., sensitivity or subgroup analyses, meta-regression), if done, indicating which were pre-specified.	Page 6 (article)
**RESULTS**			
Study selection	17	Give numbers of studies screened, assessed for eligibility, and included in the review, with reasons for exclusions at each stage, ideally with a flow diagram.	Page 7 & 8 (article): see figure (1)
Study characteristics	18	For each study, present characteristics for which data were extracted (e.g., study size, PICOS, follow-up period) and provide the citations.	Pages 9 &10 (article), and Table "1"
Risk of bias within studies	19	Present data on risk of bias of each study and, if available, any outcome level assessment (see item 12).	Page 9(article) and online only supplement (2,3)- Figures 6,7
Results of individual studies	20	For all outcomes considered (benefits or harms), present, for each study: (a) simple summary data for each intervention group (b) effect estimates and confidence intervals, ideally with a forest plot.	Page 11, 12, 13, 14 ,

			15, 16 : See figures (2,3,4,5)
Synthesis of results	21	Present results of each meta-analysis done, including confidence intervals and measures of consistency.	Page 11, 12, 13, 14, 15,16 : See figures (2,3,4,5)
Risk of bias across studies	22	Present results of any assessment of risk of bias across studies (see Item 15).	Page 9
Additional analysis	23	Give results of additional analyses, if done (e.g., sensitivity or subgroup analyses, meta-regression [see Item 16]).	Page 12 and online only supplement (4,5)- Figure “8” and Table “2”
**DISCUSSION**			
Summary of evidence	24	Summarize the main findings including the strength of evidence for each main outcome; consider their relevance to key groups (e.g., healthcare providers, users, and policy makers).	Page 17
Limitations	25	Discuss limitations at study and outcome level (e.g., risk of bias), and at review-level (e.g., incomplete retrieval of identified research, reporting bias).	Pages 20 & 21 (article)
Conclusions	26	Provide a general interpretation of the results in the context of other evidence, and implications for future research.	Page 22 (article)
**FUNDING**			
Funding	27	Describe sources of funding for the systematic review and other support (e.g., supply of data); role of funders for the systematic review.	Page 22 (article)

From: Moher D, Liberati A, Tetzlaff J, Altman DG, The PRISMA Group (2009). Preferred Reporting Items for Systematic Reviews and Meta-Analyses: The PRISMA Statement. PLoS Med 6(6): e1000097. doi:10.1371/journal.pmed1000097

For more information, visit: **www.prisma-statement.org**.

Table S3: Summary of Findings and Quality of Evidence of the Primary Outcome, RA subgroup & Respiratory Infection According to GRADE Working Group Assessment Recommendations (GRADE pro GDT).

Certainty assessment							№ of patients		Effect		Certainty
№ of studies	Study design	Study quality	Inconsistency	Indirectness	Imprecision	Other considerations	Risk of infection of MTX	Risk of infection of PBO	Relative (95% CI)	Absolute (95% CI)	
12 f	randomised trials	serious [a]	not serious [b]	not serious	not serious	none	244/670 (36.4%)	134/476 (28.2%)	**RR 1.14** (0.98 to 1.34)	**39 more per 1,000** (from 6 fewer to 96 more)	⨁⨁⨁◯ MODERATE
3 g	randomised trials	serious [c]	not serious	not serious	not serious	none	113/228 (49.6%)	59/167 (35.3%)	**RR 1.25** (1.01 to 1.56)	**88 more per 1,000** (from 4 more to 198 more)	⨁⨁⨁◯ MODERATE
5 h	randomised trials	serious [d]	not serious	not serious	not serious	none	75/347 (21.6%)	57/278 (20.5%)	**RR 0.99** (0.73 to 1.33)	**2 fewer per 1,000** (from 55 fewer to 68 more)	⨁⨁⨁◯ MODERATE

CI: Confidence interval; **RR:** Risk ratio

Explanations

a. Most information is derived from studies that are at low or unclear risk of bias, and hence the overall risk of bias across studies was considered low. Only three studies (Das et al. Singh et al, and Roychowdhury et al) had serious risk of bias, but these had no actual weight on meta-analysis. However, large amount of loss to follow up was noted in some trials (such as Strand et al, and Warren et al). Overall, evidence from RCTs here downgraded by one level.

b. Heterogeneity (inconsistency) of infectious adverse outcome estimates exists among the group of rheumatoid arthritis (RA) from one side, and non-RA diseases (such as psoriasis/psoriatic arthritis, systemic sclerosis, etc..) from the other side. However, there is a plausible biological explanation to this inconsistency

c. One trial (Strand et al) which has a significant weight in meta-analysis had a large amount of loss to follow up.

d. Even though most information is from studies that are at low or unclear risk of bias, Warren et al with relatively large weight on meta-analysis, had a large amount of loss to follow up.

f. Risk of infection of methotrexate therapy in inflammatory diseases (Primary outcome)

g. Risk of infection of methotrexate therapy in rheumatoid arthritis (RA)

h. Risk of respiratory infection of methotrexate therapy in inflammatory diseases

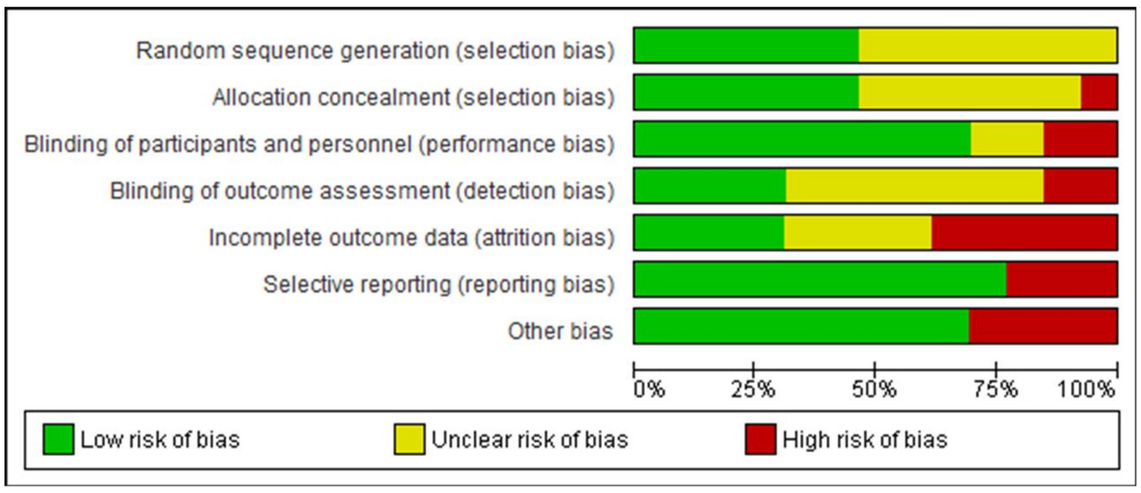

Figure S1. Risk of bias graph: review authors' judgements about each risk of bias item presented as percentages across all included studies.

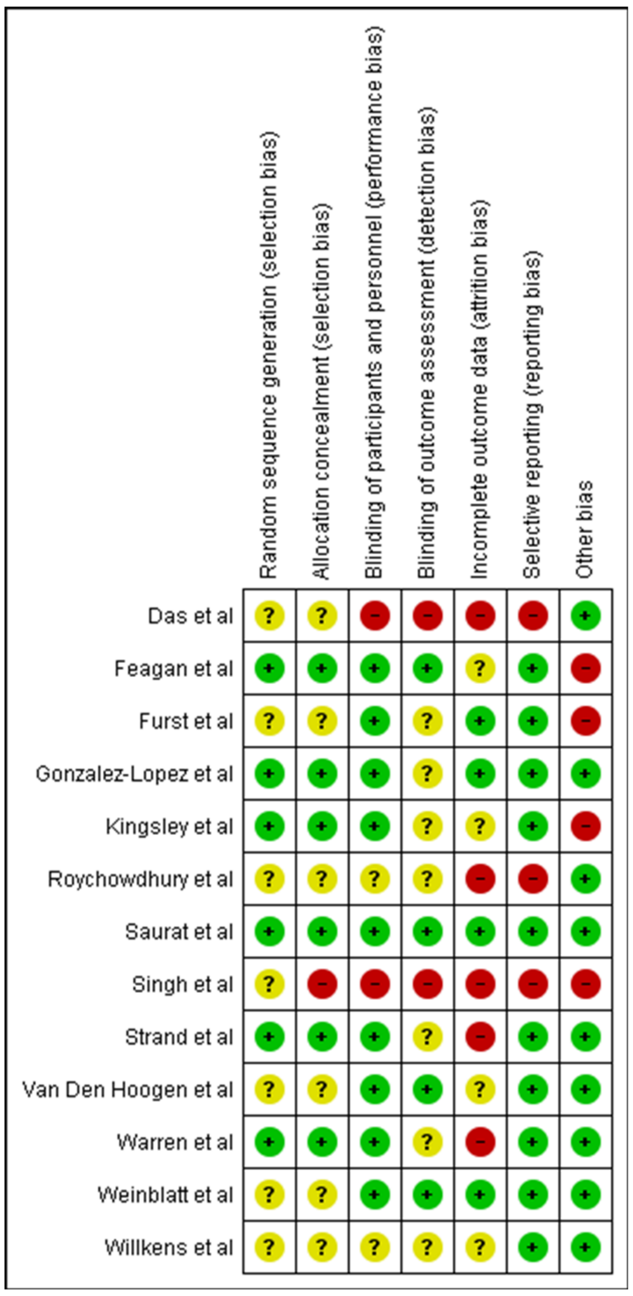

Figure S2. Risk of bias summary: Review authors' judgements about each risk of bias item for each included study.

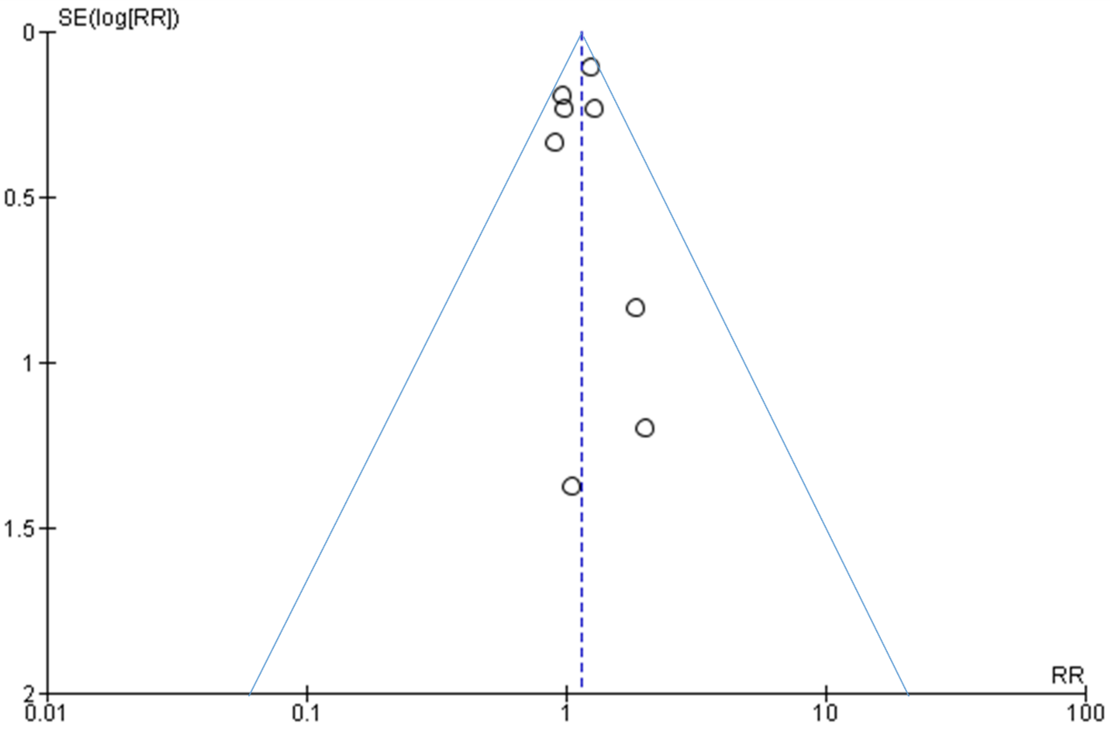

Figure S3. Funnel plot of risk of infection with methotrexate therapy in inflammatory diseases to assess publication bias.
